# Neutrophil Percentage/Albumin Ratio as an Independent Predictor of the No-Reflow Phenomenon in Patients with ST-Elevation Myocardial Infarction Undergoing Primary Percutaneous Coronary Intervention

**DOI:** 10.3390/diagnostics15202609

**Published:** 2025-10-16

**Authors:** Ozkan Yavcin, Yucel Yilmaz

**Affiliations:** 1 Department of Cardiology, Ministry of Health, Elazig Fethi Sekin City Hospital, 23280 Elazig, Türkiye; ozkanycn@hotmail.com; 2 Department of Cardiology, Kayseri City Training and Research Hospital, University of Health Sciences, 38010 Kayseri, Türkiye

**Keywords:** neutrophil percentage/albumin ratio, ST-elevation myocardial infarction, no-reflow phenomenon, inflammation

## Abstract

**Objectives:** Despite achieving a high rate of revascularization in epicardial coronary arteries with primary percutaneous coronary intervention (pPCI), suboptimal coronary reperfusion is encountered in more than half of patients. This condition, termed the ‘no-reflow phenomenon’ (NRP), has been associated with ventricular arrhythmias, left ventricular dysfunction, impaired ventricular remodeling, myocardial reinfarction, and increased mortality. The neutrophil percentage/albumin ratio (NPAR) has been associated with the severity and prognosis of cardiovascular patients. The aim of this study is to investigate the relationship between NRP and NPAR in patients undergoing pPCI with a diagnosis of ST-elevation myocardial infarction (STEMI). **Methods:** A total of 758 patients diagnosed with STEMI and undergoing pPCI were included in this study. A total of 105 patients were detected to have NFP during pPCI (13.8%). Slow flow, such as thrombolysis in myocardial infarction (TIMI) 0, 1, or 2, observed in the distal part of the coronary artery after pPCI, was operationally defined as NRP. Reflow was defined as TIMI 3. NPAR was obtained by dividing the neutrophil percentage by albumin. **Results:** Statistically, there was a significant difference between the groups in terms of mean age, body mass index (BMI), and left ventricular ejection fraction (LVEF), which were higher in the NRP group [54 (45–62) vs. 60 (53–67), 26.5 (23.6–30.8) vs. 28.4 (26–31), and 39.2 ± 6.9 vs. 31.8 ± 5.1; *p* < 0.001, for all]. When laboratory parameters were examined between the two groups, white blood cell (WBC) count, neutrophil count, neutrophil percentage, C-reactive protein (CRP), neutrophil/lymphocyte ratio (NLR), NPAR and CRP/albumin ratio (CAR) levels were found to be statistically significantly higher in the patient group with NRP (*p* < 0.05). Multivariate analysis identified NPAR as an independent predictor of NRP (5.482, 3.254–9.234, *p* < 0.001). ROC analysis demonstrated that the best cutoff value of 18.45 for NPAR was to predict NRP with 80% sensitivity and 75% specificity (area under ROC curve = 0.826 (95% CI: 0.770–0.881), *p* < 0.001). **Conclusions:** We found that NPAR levels at admission were independently associated with the development of NRP pPCI in patients with STEMI.

## 1. Introduction

Cardiovascular disease (CVD)-related deaths rank among the leading causes of death overall, and myocardial infarctions (MIs) are the most significant cause of cardiovascular deaths [1,2]. ST-segment elevation myocardial infarction (STEMI) is one of the most serious forms of coronary artery disease (CAD), typically characterized by acute onset and high mortality [3]. First performed in 1979 by Grüntzig et al. [4], primary percutaneous coronary intervention (pPCI) is recommended as a class I therapeutic approach for managing STEMI treatment in the latest guidelines published by the European Society of Cardiology [5]. Despite achieving a high rate of revascularization in epicardial coronary arteries with pPCI, more than half of patients experience suboptimal coronary reperfusion [6]. This phenomenon, thought to stem from structural and functional changes in coronary microcirculation, is termed the ‘no-reflow phenomenon’ [6]. The no-reflow phenomenon (NRP) has been associated with various adverse outcomes, including ventricular arrhythmias, left ventricular dysfunction, impaired ventricular remodeling, myocardial reinfarction and increased mortality [7,8].

It has been suggested that the NRP is associated with ischemic damage, thromboembolism, leukocyte activation, distal atherosclerosis, reperfusion injury and oxidative stress [9]. Progressive leukocyte activation is also important for NRP development. Engler et al. [10] reported that the leukocyte frequency in myocardial capillaries with NRP was ten times higher than that in adequately perfused myocardium. Researchers have suggested that capillary leukocyte obstruction has a significant effect on NRP development in the first hour of myocardial ischemia and is less common in neutropenic patients [11,12]. Furthermore, the width of the NRP zone was shown to be reduced in the neutropenic persons [12]. Capillary obstruction by neutrophils not only creates a mechanical obstruction to flow but also causes increased distal obstruction, degeneration of smooth muscle and endothelial cells, and destabilization of the atherosclerotic plaque through released mediators [11,13,14]. Furthermore, it has been suggested that induced neutrophil extracellular traps (NETs) may exacerbate endothelial damage [15]. The increased inflammatory response leads to platelet aggregation and subsequent coronary thrombus formation [16].

Different pathophysiological mechanisms make determining who will develop NRP, its progression, and its treatment more complex and challenging. Furthermore, even if NRP is treated, morbidity/mortality cannot be guaranteed. Therefore, it is important to determine the risk of NRP development, its complications, and personalized treatment pathways to ensure the diagnosis/treatment management of these patients. Inflammatory biomarkers have increasingly been evaluated in recent studies due to their affordability, simplicity, reproducibility, non-invasiveness, and ease of use in large populations, including CAD patients with NRP [17,18,19,20,21].

Studies on numerous inflammatory biomarkers, including the neutrophil/lymphocyte ratio (NLR), lymphocyte/monocyte ratio (LMR), and systemic immune-inflammation index (SII), are available and appear likely to enter routine laboratory use in the not-too-distant future [22,23,24,25,26]. The neutrophil percentage/albumin ratio (NPAR), which is obtained by dividing the neutrophil percentage by albumin and has recently become more popular, has been studied in CVD at an earlier stage than other biomarkers [27,28,29]. This biomarker contains two parameters, neutrophils, a cell group that closely reflects the host’s inflammatory state, and albumin, which reflects anti-inflammatory/antioxidant status, that appear to buffer each other. Therefore, it may provide a new biomarker that is more sensitive, stable, and reliable than previous biomarkers (NLR, PLR, CAR, etc.) used individually [30,31,32,33,34,35].

However, to date, no study has specifically evaluated the relationship between NRP and pPCI in NPAR and STEMI patients. The aim of this study is to investigate the relationship between NRP and NPAR in patients diagnosed with STEMI and admitted to the coronary care unit (CCU) who underwent pPCI and to evaluate our hypothesis that this relationship may contribute to risk stratification methods in these patients.

## 2. Material and Methods

### 2.1. Criteria for Participant Inclusion

Between 2014 and 2022, 758 patients aged 18–80 years who were admitted to our hospital, diagnosed with STEMI according to the ESC guidelines, and underwent pPCI were included in this study. This study was designed as a single-center retrospective observational cohort study.

We established the following exclusion criteria: patients with unstable vital signs such as heart rate and/or blood pressure, hospital admission more than 12 h after symptom onset, patients who have received fibrinolytic therapy within the last 24 h, previous coronary artery bypass grafting (CABG), history of acute coronary syndrome and/or PCI within the last 3 months, heart failure, hematological diseases, malignancy, active infection or documented systemic inflammatory disease, known malignancies, or terminal liver or renal failure.

Among all applicants, 29 patients were excluded from the study due to use of anti-inflammatory drugs, 78 due to excessively high C-reactive protein (CRP) levels, 176 due to incomplete medical records and test results, 146 due to unstable vital signs (killip 3–4, cardiogenic shock, pulmonary edema, etc.), and 279 due to cardiovascular reasons (presentation more than 12 h late, received thrombolytic therapy, had CABG, and had acute coronary event and/or PCI within the last 3 months).

### 2.2. Data Collection and Definitions

The variables included in this study (laboratory findings, medical history, and physical examinations) were obtained from archival records and/or computer records. We also recorded classic risk factors for CVD [age, gender, diabetes mellitus (DM), hypertension (HT), and dyslipidemia]. Participants whose complete medical records were available were included in this study.

DM diagnoses of patients included in the study were defined according to the 2019 American Diabetes Association Standards of Medical Care Guidelines, while HT diagnoses were defined according to the 2018 ESC/ESH Guidelines for the Management of Arterial Hypertension [36,37]. Smokers were defined as those who had smoked regularly for at least 1 year. For the diagnosis of dyslipidemia, patients were defined as having low-density lipoprotein cholesterol (LDL-C) levels > 130 mg/dL at admission and/or having a previous diagnosis of dyslipidemia and/or receiving lipid-lowering therapy [38].

### 2.3. Definition of the No-Reflow Phenomenon

Following pPCI, the no-reflow phenomenon is defined as inadequate myocardial perfusion in a specific segment of the coronary artery despite the absence of mechanical causes such as coronary dissection, vasospasm, or thrombus formation. Thrombolysis in myocardial infarction (TIMI) blood flow grades are classified as follows: Grade 0 (no perfusion), Grade 1 (partial contrast passage from the occluded segment without filling the distal vessels), Grade 2 (complete filling of the distal artery with contrast, but with slower than normal flow), and Grade 3 (normal perfusion with complete and rapid filling and washout of the distal vessels with contrast). In this study, slow flow such as TIMI 0, 1, or 2 in the distal coronary artery after pPCI was operationally defined as NRP. Reflow was defined as TIMI 3 [39,40]. In addition, a myocardial blush score of at least 2 was also accepted.

Myocardial blush grading (MBG) levels after pPCI are categorized as follows: level 0 (no contrast enhancement), level 1 (minimal contrast enhancement), level 2 (moderate but below normal contrast enhancement), and level 3 (normal contrast enhancement).

Intra- and inter-observer variability for assessing angiographic TIMI flow grading were 2% and 3%, respectively.

### 2.4. Coronary Diagnostic Imaging and Percutaneous Intervention

Baseline coronary angiography was performed using standard techniques. All primary pPCI procedures were performed using radial or femoral arterial access. Cineangiography analysis was performed using the Artis Zee Floor workstation (Siemens Medical Solutions, Erlangen, Germany). During pPCI, non-fractionated heparin was administered as a standard intravenous bolus and, if necessary, with additional doses (70–100 U/kg), thereby maintaining activated clotting time for more than 250 s. All coronary angiograms were recorded in digital format for quantitative analysis. Immediately after coronary angiography, stenting of the artery associated with the infarction was performed in appropriate cases (with drug-eluting stents). According to institutional protocol, the use of glycoprotein IIb/IIIa inhibitors was left to the operator’s discretion. Two experienced cardiologists, unaware of all clinical data, analyzed the thrombolysis (TIMI) flow grade in myocardial infarction before and after the intervention. In cases of disagreement, a third cardiologist also reviewed the coronary angiograms and reached a consensus.

### 2.5. Medication Protocols

All patients received a loading dose of 300 mg of aspirin and antiplatelet therapy (600 mg of clopidogrel or 180 mg of ticagrelor or 60 mg of prasugrel) at the operator’s discretion during STEMI diagnosis. Perioperative basic medications followed clinical guidelines [5]. Other oral medication therapies were determined by the CCU cardiologists and adjusted according to the STEMI guidelines [5]. The use of glycoprotein IIb/IIIa inhibitors was planned by the operator according to the patient’s clinical condition. The use of other peri-procedural medications, such as intracoronary nitroglycerin and adenosine, was also left to the operator’s discretion.

### 2.6. Biochemical Testing and Cardiac Ultrasound Analysis

Venous blood samples were collected from all patients using vacuum blood collection tubes from the antecubital region on the morning after admission and before coronary angiography after a period of fasting. All routine biochemical tests, including glucose, high-sensitivity C-reactive protein (hs-CRP), lipid profile, and serum creatinine (SCr), were performed on an autoanalyzer (COBAS^®^ c701, Roche Diagnostics, Mannheim, Germany). Hematological parameters were measured using an automated hematology analyzer system (Sysmex K-1000 Hematology Analyzer, Kobe, Japan). The neutrophil/lymphocyte ratio (NLR) was calculated by dividing the neutrophil count by the lymphocyte count, and the CRP/albumin ratio (CAR) was calculated by dividing the CRP levels by the albumine levels. NPAR was obtained by dividing the neutrophil percentage by albumin

All echocardiographic measurements were performed within 24 h after the procedures using a GE ViVidE5 ultrasound device (GE Healthcare, Piscataway, NJ, USA/New York City, NY, USA) with a 3.5 MHz transducer. Left ventricular ejection fraction (LVEF) was measured using the Simpson method according to the recommendations of the American Society of Echocardiography.

## 3. Statistical Analysis

Statistical analyses were performed using SPSS Statistics Package for Windows version 21.0 (SPSS Inc., Chicago, IL, USA). Continuous variables with normal distribution were analyzed using the Shapiro–Wilk test. Means and standard deviations of continuous data were evaluated and recorded. The distribution of continuous variables between groups was determined using Student’s *t*-test or Mann–Whitney U test. Variability between groups was determined using the *t*-test. The chi-square test was used for categorical variables and was calculated as percentages. The relationship between variables was analyzed using Pearson correlation analysis. We also used logistic regression analyses to observe the relationship between NRP and the effect of the primary variable and other variables possibly acting as confounders (age in years; neutrophil percentage; CAD; presence of hypertension; presence of DM; etc.). All factors with a significance of *p* < 0.05 were entered into stepwise multivariate logistic regression analysis. A *p* value less than 0.05 was considered significant.

The Hosmer–Lemeshow test statistic was 11.174 (df = 6; *p* = 0.192), which indicated a good fit between observed and predicted outcomes.

## 4. Results

A total of 105 patients detected to have coronary NRP during coronary angiography and 653 control subjects with coronary normal flow after pPCI procedure were included in the study.

There were 24.8% women in the NRP group and 29% women in the coronary normal flow group, and there was no statistically significant difference between the two groups (*p* < 0.05). Statistically, a significant difference was found between the groups in terms of mean age, body mass index (BMI), and LVEF, which were higher in the NRP group [54 (45–62) vs. 60 (53–67), 26.5 (23.6–30.8) vs. 28.4 (26–31), and 39.2 ± 6.9 vs. 31.8 ± 5.1; *p* < 0.001, for all]. When considering the presence of DM, dyslipidemia, which is one of the CAD risk factors, and the presence of CAD according to the anamnesis taken, there were no significant differences between the two groups (*p* > 0.05) (Table 1).

There was no significant difference between patients with NRP and normal coronary flow groups in terms of hemodynamic parameters, heart rate, systolic, and diastolic blood pressure.

When the laboratory analyses are evaluated, some parameters in terms of biochemical parameters reveal that there is no significant difference between the groups, such as total cholesterol, low-density lipoprotein cholesterol (LDL), high-density lipoprotein (HDL-C) cholesterol, triglyceride levels, glucose levels, and glomerular filtration rate levels (*p* > 0.05) (Table 1). However, albumin values were found to be significantly lower in the NRP group than in the normal coronary flow (Table 1).

When the laboratory parameters were examined between the two groups, no significant difference was found between hemoglobin, platelet, and lymphocyte counts (*p* > 0.05), while a significant difference was found between WBC levels, neutrophil numbers, and neutrophil percentage (*p* < 0.001). Additionally, CRP levels [(3.2 (2.4–6.1) vs. 5 (3.6–8.2), *p* < 0.001] along with NLR [2.1 (1.5–3) vs. 4.6 (2.9–9.6), *p* < 0.001], NPAR [16.32 ± 4.14 vs. 21.01 ± 3.65, *p* < 0.001], and CAR levels [0.75 (0.59–1.7) vs. 1.1 (0.74–2.1), *p* < 0.001] were statistically significantly higher in the NRP group.

Angiographic data results are shown in Table 2. No difference was found between groups in terms of vessel type associated with infarction, stent length/diameter used, and whether predilation was performed or not (*p* > 0.05).

Risk factors thought to affect NRP were evaluated using univariate and then multivariate analyses (Table 3). Age, HT, DM, dyslipidemia, BMI, LVEF, albumin, WBC, neutrophil, CRP, NLR, NPAR, and CAR, which were statistically significant in the univariate analysis, were included in the multivariate analysis. Age (Odds Ratio (OR) 1.108, 95% confidence e interval (CI): 1.072–1.144, *p* < 0.001), BMI (OR; 1.016, 95% CI; 1.011–1.148, *p* = 0.022), NLR (OR; 1.158, 95% CI; 1.074–1.249, *p *< 0.001), NPAR (OR; 5.482, 95% CI; 3.254–9.234, *p* < 0.001), and CAR (OR; 1.344, 95% CI; 1.024–1.763, *p* = 0.033) were found to be independent predictors of NRP.

**Figure 1:**
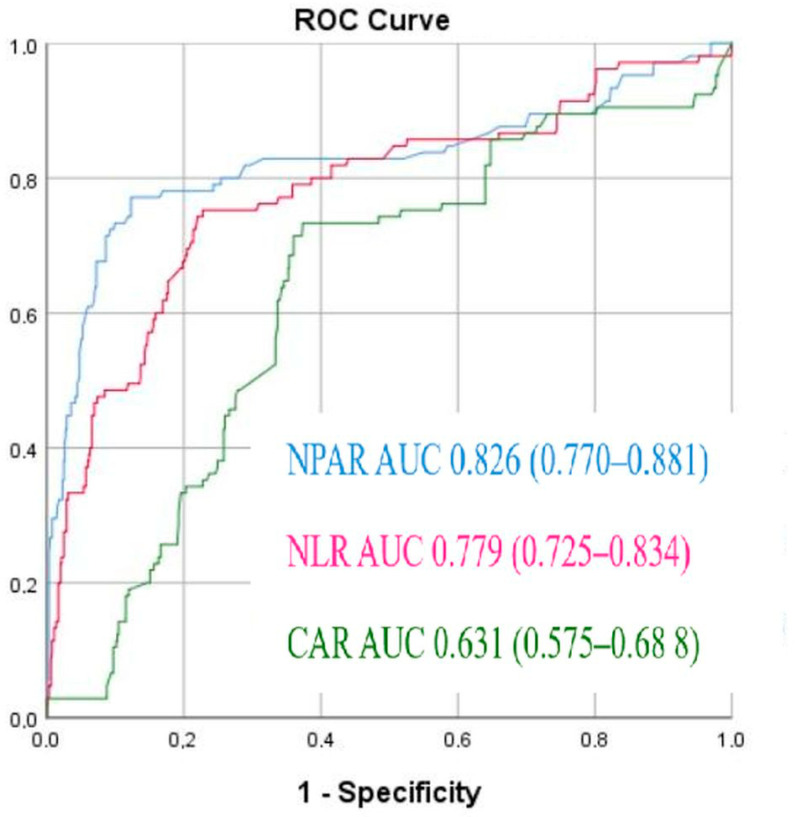
Receiver operating characteristic (ROC) curves for the Neutrophil percentage/albumin ratio (NPAR), C-reactive protein/albumin ratio, and neutrophil-to-lymphocyte ratio (NLR) to predict no-reflow phenomenon.

ROC analysis demonstrated that the optimal cutoff value of 18.45 for NPAR predicted NRP with 80% sensitivity and 75% specificity (area under the ROC curve = 0.826 (95% CI: 0.770–0.881), *p* < 0.001) (Figure 1).

## 5. Discussion

This study investigated the effect of NPAR values on the development of NRP, which is known to negatively impact cardiac morbidity/mortality, and found results suggesting that NPAR may be effective in predicting NRF. In this regard, our study is the first of its kind.

The etiology and pathogenesis of post-pPCI NRP are complex, and while distal thromboembolism is considered the most important cause, other factors such as ischemic and/or reperfusion injury, leukocyte invasion, and coronary microcirculatory damage also play a role [41,42]. Male gender, advanced age, family history of CAD, DM, smoking, HT and delay in PCI are considered potential risk factors [43]. Furthermore, inflammatory mediators cause endothelial dysfunction, which promotes leukocyte adhesion and infiltration into the endothelium. Ultimately, this can lead to insufficient blood flow due to microvascular occlusion. The resulting inflammatory process can further stress the endothelium, causing edema in the endothelium of the capillaries, leading to microvascular obstruction and increasing the risk of NRP [44]. Considering all these etiology and pathogenesis, while the predictive value of inflammatory response markers for NRP formation after pPCI in STEMI patients remains a subject of debate, it is not unreasonable to consider inflammation as a contributing factor.

Although there is much speculation about the mechanisms underlying the development of NRP, an increasing number of studies suggest that inflammation/increased inflammatory response plays a role in the development of NRP. Tian et al. [45] reported in their 2017 study that high neutrophil counts may be associated with NRP. Vakili et al. [17] reported in another study conducted in the same year that there may be an independent relationship between PLR and NLR and NRP. Refaat et al. [18] claimed in their 2021 study that elevated CAR and atherogenic index of plasma (AIP) levels may predict the development of NRP. In 2024, Qu et al. [19] claimed that the triglyceride–glucose (TyG) index is a strong predictor of NRP development in STEMI patients, could be useful for risk classification, and could enable the adaptation of preventive strategies. Esenboğa et al. [20] in 2022 with SII and Bayramoğlu et al. [21] in 2023 with pan-immune-inflammation value (PIV) reported that there may be a relationship between these inflammatory parameters and NRP. In this study, levels of CRP, NLR, CAR, and NPAR, considered biomarkers of inflammation, were found to be higher in those who developed NRP than in those who did not. These results are consistent with the results of the studies we reviewed above and led us to conclude that they may be independently associated with NRP.

Although NPAR is a much newer marker, it is increasingly being used in studies involving patients with cardiovascular disease, particularly those with CAD. Yin et al. [46] showed that there may be a relationship between the slow coronary flow phenomenon and NPAR. In two other studies specifically on NPAR, Karasu et al. [47] reported its relationship with CAD severity in patients who had experienced NSTEMI, while Zhao et al. [48] reported its relationship with CAD severity, particularly in CAD patients with chronic renal failure. Serhatlioglu et al. [49] claimed that NPAR could be used to predict AF developing in the early period after CABG surgery. Cui et al. [27] found an independent association between NPAR and in-hospital mortality in patients with ST-elevation myocardial infarction (STEMI). Karaca et al. [50] claimed that NPAR is a newly identified promising inflammatory biomarker for predicting one-year major adverse cardiac and cerebrovascular events (MACCE) in non-ST elevation myocardial infarction (NSTEMI) patients undergoing revascularization therapy. In this study, NPAR levels were observed to be higher in patients who developed NRP after pPCI in STEMI patients, and results suggest that it can independently predict the risk of developing NPR. These results suggest that NPAR may be useful in predicting the risk of developing NPR and that the inflammatory status assessed during hospitalization should not be overlooked in predicting NRP, a significant complication after pPCI.

NPAR can represent the balance between the two systems and can be considered a measure of the response to stress and/or systemic inflammation. As is well known, increased neutrophils represent the activation of inflammation, while albumin is a negative acute phase reactant. When neutrophils are activated in response to tissue damage, their tropism for inflamed tissues emerges, performing tasks such as the release of proteolytic enzymes, arachidonic acid derivatives, and superoxide radicals [34,35]. Additionally, the aggregation that occurs between platelets and neutrophils leads to blockage in capillaries and mechanically impedes blood flow. Vasoconstrictor mediators released from physiologically or pathologically altered/dysfunctional cells (endothelial cells, neutrophils, and platelets) affect coronary microcirculation [51]. Albumin, in addition to its primary roles of transporting physiologically active substances and buffering pH, serves as an indicator of the body’s nutritional status. Perhaps more importantly, it performs functions such as exhibiting anti-inflammatory and antioxidant properties [30]. Albumin is a negative acute phase protein, and a decrease in albumin levels leads to increased blood viscosity, endothelial dysfunction, platelet aggregation, increased oxidation of low-density lipoprotein cholesterol (LDL-C), and consequently increased foam cell formation and inflammation [31,32,33,52]. Low albumin levels may cause a decrease in antioxidant defenses and increased susceptibility to oxidative stress [53].

In conclusion, NPAR, as a new index, is simple, readily available, cost-effective, and readily applicable in clinical practice. Based on these results, it is reasonable to speculate that STEMI patients with high baseline NPAR values may have a relatively higher pre-procedural risk of developing NRP. NPAR could be added to the risk stratification of NRP development. Perhaps in the future, considering the higher risk of NRP in STEMI patients with high NPAR values, preventive measures (more aggressive anticoagulation, antiplatelet therapy, strict blood pressure control, contrast medium volume, etc.) could be considered before pPCI and closer follow-up after pPCI could be considered. However, evaluation of these recommendations requires larger, multicenter, prospective studies.

## 6. Limitations

Some limitations of this study can be summarized as follows: it is a single-center, retrospective design and includes a relatively small number of patients. It does not include pre-hospital and post-discharge laboratory data. This limits the ability to obtain information on the long-term effects of NPAR. Because the study was not conducted at the cellular/tissue level, microscopic effects cannot be commented on. Furthermore, the statistical tests we used to obtain the study results do not inherently imply causality.

## Figures and Tables

**Table 1:** Baseline characteristics of study groups.

	Normal-Reflow *n* = 653	No-Reflow *n* = 105	*p* Value
Age (year)	54 (45–62)	60 (53–67)	<0.001
Male (*n*, %)	461 (71%)	79 (75.2%)	0.33
Hypertension (*n*, %)	362 (55.4)	64 (60.1%)	0.089
DM (*n*, %)	258 (39.5%)	42 (40%)	0.815
Dyslipidemia (*n*, %)	87 (13.3%)	18 (17.1%)	0.318
CAD (*n*, %)	124 (19%)	26 (24.7%)	0.655
Smokers (*n*, %)	315 (48.2%)	49 (46.6%)	0.354
LVEF (%)	39.2 ± 6.9	31.8 ± 5.1	<0.001
Killip III–IV (*n*, %)	29 (4.4%)	9 (8.2%)	0.062
BMI (kg/m^2^)	26.5 (23.6–30.8)	28.4 (26–31)	<0.001
Glomerular filtration rate (mL/min/1.73 m^2^)	85.29 ± 39.4	81.9 ± 37.2	0.17
Serum Glucose (mg/dL)	109.29 ± 68.6	129.5 ± 34.5	0.144
Total cholesterol (mg/dL)	156.3 (132–179)	175 (141–210)	0.291
High-density lipoprotein cholesterol (mg/dL)	39 (29–47)	37 (31–45)	0.713
Low-density lipoprotein cholesterol (mg/dL)	101 (79–135)	106 (82–176)	0.645
Triglyceride (mg/dL)	129 (83–211)	144 (91–233)	0.536
Albumin (mg/dL)	4.2 (3.5–4.4)	3.9 (3.5–4.1)	0.021
Hemoglobin (g/L)	14.2 (13.3–15.2)	14.3 (12.9–15.1)	0.883
Platelet (10^3^/μL)	220 (188–263)	227 (187–257)	0.716
WBC (10^3^/μL)	7.4 (5.8–8.9)	12.3 (9.6–16.9)	<0.001
Neutrophil (10^3^/μL)	4.1 (3.3–6.9)	7 (4.5–13.5)	<0.001
Neutrophil percentage (%)	67.2 (45.2–75.3)	81.9 (66.9–89.1)	<0.001
Lymphocyte (10^3^/μL)	1.8 (1.3–2.5)	1.7 (1.3–2.3)	0.168
CRP (mg/dL)	3.2 (2.4–6)	5 (3.6–8)	<0.001
NLR	2.1 (1.5–3)	4.6 (2.9–9.6)	<0.001
NPAR	16.32 ± 4.14	21.01 ± 3.65	<0.001
CAR	0.75 (0.59–1.7)	1.1 (0.74–2.1)	<0.001

Abbreviations: DM: Diabetes Mellitus, CAD: Coronary arterial disease, BMI: Body mass index, LVEF: Left ventricular ejection fraction, WBC: white blood cell, CRP: C-reactive protein, NLR: Neutrophil/Lymphocyte ratio, CAR: CRP/Albumin ratio, NPAR: Neutrophil percentage/albumin ratio. Values are mean ± SD for normally distributed variables, medians [IQRs] for skewed variables or *n* (%) for categorical or discrete variables.

**Table 2:** Angiographic data.

Infarct Related Artery			
LAD	76 (49%)	113 (45%)	0.322
RCA	42 (27%)	71 (28%)	0.843
CX	35 (23%)	67 (26%)	0.334
Coronary artery involvement			
Stent length (mm)	21.1 ± 5.4	20.6 ± 6.7	0.139
Stent diameter (mm)	2.54 ± 0.85	2.61 ± 0.73	0.380
Predilation	103 (67%)	148 (60%)	0.113

Abbreviations: LAD: Left anterior descending, CX: Circumflex artery, RCA: Right coronary artery.

**Table 3:** Effects of multiple variables on the no-reflow phenomenon in univariate and multivariate logistic regression analyses.

	Univariate Analysis			Multivariate Analysis		
Variables	Unadjusted OR	95% CI	*p* Value	Adjusted OR	95% CI	*p* Value
Age	1.057	1.036–1.079	<0.001	1.108	1.072–1.144	<0.001
BMI	1.063	1.018–1.111	0.006	1.016	1.011–1.148	0.022
LVEF	0.592	0.345–0.878	0.026			
Albumin	0.488	0.303–0.786	0.003			
WBC	1.403	1.312–1.501	<0.001			
Neutrophil	1.501	1.386–1.625	<0.001			
Neutrophil percentage	3.625	2.982–5.193	<0.001			
CRP	1.068	1.020–1.119	0.005			
NLR *	1.324	1.245–1.408	<0.001	1.158	1.074–1.249	<0.001
NPAR *	4.622	3.452–6.190	<0.001	5.482	3.254–9.234	<0.001
CAR	1.369	1.137–1.647	0.001	1.344	1.024–1.763	0.033

Abbreviations: BMI: Body mass index, LVEF: Left ventricular ejection fraction, * These parameters are not entered into the model with each other to avoid multicollinearity.

## Data Availability

The original contributions presented in this study are included in the article. Further inquiries can be directed to the corresponding author.

## References

[1] Roth G.A., Mensah G.A., Johnson C.O., Addolorato G., Ammirati E., Baddour L.M., Barengo N.C., Beaton A.Z., Benjamin E.J., Benziger C.P. (2020). Global Burden of Cardiovascular Diseases and Risk Factors, 1990–2019: Update from the GBD 2019 Study. J. Am. Coll. Cardiol..

[2] Vaduganathan M., Mensah G.A., Turco J.V., Fuster V., Roth G.A. (2022). The Global Burden of Cardiovascular Diseases and Risk: A Compass for Future Health. J. Am. Coll. Cardiol..

[3] Poudel I., Tejpal C., Rashid H., Jahan N. (2019). Major Adverse Cardiovascular Events: An Inevitable Outcome of ST-Elevation Myocardial Infarction? A Literature Review. Cureus.

[4] Grüntzig A.R., Senning A., Siegenthaler W.E. (1979). Nonoperative Dilatation of Coronary-Artery Stenosis: Percutaneous Transluminal Coronary Angioplasty. N. Engl. J. Med..

[5] Ibanez B., James S., Agewall S., Antunes M.J., Bucciarelli-Ducci C., Bueno H., Caforio A.L.P., Crea F., Goudevenos J.A., Halvorsen S. (2018). 2017 ESC Guidelines for the Management of Acute Myocardial Infarction in Patients Presenting with ST-Segment Elevation: The Task Force for the Management of Acute Myocardial Infarction in Patients Presenting with ST-Segment Elevation of the European Society of Cardiology (ESC). Eur. Heart J..

[6] Pelliccia F., Niccoli G., Zimarino M., Andò G., Porto I., Calabrò P., De Rosa S., Gragnano F., Piccolo R., Moscarella E. (2023). Pathophysiology and Treatment of the No-Reflow Phenomenon in ST-Segment Elevation Myocardial Infarction: Focus on Low-Dose Fibrinolysis during Primary Percutaneous Intervention. Rev. Cardiovasc. Med..

[7] Niccoli G., Burzotta F., Galiuto L., Crea F. (2009). Myocardial No-Reflow in Humans. J. Am. Coll. Cardiol..

[8] Gerber B.L., Rochitte C.E., Melin J.A., McVeigh E.R., Bluemke D.A., Wu K.C., Becker L.C., Lima J.A. (2000). Microvascular Obstruction and Left Ventricular Remodeling Early After Acute Myocardial Infarction. Circulation.

[9] Annibali G., Scrocca I., Aranzulla T.C., Meliga E., Maiellaro F., Musumeci G. (2022). “No-Reflow” Phenomenon: A Contemporary Review. J. Clin. Med..

[10] Engler R.L., Schmid-Schönbein G.W., Pavelec R.S. (1983). Leukocyte Capillary Plugging in Myocardial Ischemia and Reperfusion in the Dog. Am. J. Pathol..

[11] Engler R.L., Dahlgren M.D., Morris D.D., Peterson M.A., Schmid-Schönbein G.W. (1986). Role of Leukocytes in Response to Acute Myocardial Ischemia and Reflow in Dogs. Am. J. Physiol..

[12] Litt M.R., Jeremy R.W., Weisman H.F., Winkelstein J.A., Becker L.C. (1989). Neutrophil Depletion Limited to Reperfusion Reduces Myocardial Infarct Size after 90 Minutes of Ischemia. Evidence for Neutrophil-Mediated Reperfusion Injury. Circulation.

[13] Mazzoni M.C., Borgström P., Warnke K.C., Skalak T.C., Intaglietta M., Arfors K.E. (1995). Mechanisms and Implications of Capillary Endothelial Swelling and Luminal Narrowing in Low-Flow Ischemias. Int. J. Microcirc. Clin. Exp..

[14] Duilio C., Ambrosio G., Kuppusamy P., DiPaula A., Becker L.C., Zweier J.L. (2001). Neutrophils Are Primary Source of O2 Radicals during Reperfusion after Prolonged Myocardial Ischemia. Am. J. Physiol. Heart Circ. Physiol..

[15] Ge L., Zhou X., Ji W.-J., Lu R.-Y., Zhang Y., Zhang Y.-D., Ma Y.-Q., Zhao J.-H., Li Y.-M. (2015). Neutrophil Extracellular Traps in Ischemia-Reperfusion Injury-Induced Myocardial No-Reflow: Therapeutic Potential of DNase-Based Reperfusion Strategy. Am. J. Physiol. Heart Circ. Physiol..

[16] Golino P., Maroko P.R., Carew T.E. (1987). Efficacy of Platelet Depletion in Counteracting the Detrimental Effect of Acute Hypercholesterolemia on Infarct Size and the No-Reflow Phenomenon in Rabbits Undergoing Coronary Artery Occlusion-Reperfusion. Circulation.

[17] Vakili H., Shirazi M., Charkhkar M., Khaheshi I., Memaryan M., Naderian M. (2017). Correlation of Platelet-to-Lymphocyte Ratio and Neutrophil-to-Lymphocyte Ratio with Thrombolysis in Myocardial Infarction Frame Count in ST-Segment Elevation Myocardial Infarction. Eur. J. Clin. Investig..

[18] Refaat H., Tantawy A., Gamal A.S., Radwan H. (2021). Novel Predictors and Adverse Long-Term Outcomes of No-Reflow Phenomenon in Patients with Acute ST Elevation Myocardial Infarction Undergoing Primary Percutaneous Coronary Intervention. Indian Heart J..

[19] Qu Z., Guan X. (2024). Predictive Value of the Triglyceride-Glucose Index for No-Reflow Phenomenon after Percutaneous Coronary Intervention in Patients with Acute ST-Segment Elevation Myocardial Infarction Complicated by Metabolic Syndrome. Am. J. Transl. Res..

[20] Esenboğa K., Kurtul A., Yamantürk Y.Y., Tan T.S., Tutar D.E. (2022). Systemic Immune-Inflammation Index Predicts No-Reflow Phenomenon after Primary Percutaneous Coronary Intervention. Acta Cardiol..

[21] Bayramoğlu A., Hidayet Ş. (2023). Association between Pan-Immune-Inflammation Value and No-Reflow in Patients with ST Elevation Myocardial Infarction Undergoing Percutaneous Coronary Intervention. Scand. J. Clin. Lab. Investig..

[22] Soylu K., Gedikli Ö., Dagasan G., Aydin E., Aksan G., Nar G., İnci S., Yilmaz Ö. (2015). Neutrophil-to-Lymphocyte Ratio Predicts Coronary Artery Lesion Complexity and Mortality after Non-ST-Segment Elevation Acute Coronary Syndrome. Rev. Port. Cardiol..

[23] de Liyis B.G., Ciaves A.F., Intizam M.H., Jusuf P.J., Artha I.M.J.R. (2023). Hematological Biomarkers of Troponin, Neutrophil-to-Lymphocyte Ratio, and Monocyte-to-Lymphocyte Ratio Serve as Effective Predictive Indicators of High-Risk Mortality in Acute Coronary Syndrome. Biomedicine.

[24] Orhan A.L., Şaylık F., Çiçek V., Akbulut T., Selçuk M., Çınar T. (2022). Evaluating the Systemic Immune-Inflammation Index for in-Hospital and Long-Term Mortality in Elderly Non-ST-Elevation Myocardial Infarction Patients. Aging Clin. Exp. Res..

[25] Serhatlioglu F., Cetinkaya Z., Yilmaz Y. (2024). The Role of Glucose-Lymphocyte Ratio in Evaluating the Severity of Coronary Artery Disease. J. Clin. Med..

[26] Duran M., Uysal O.K., Günebakmaz O., Yılmaz Y., Akın F., Baran O., Inanç M.T., Eryol N.K., Ergin A., Oğuzhan A. (2013). Increased Red Cell Distribution Width Level Is Associated with Absence of Coronary Collateral Vessels in Patients with Acute Coronary Syndromes. Turk. Kardiyol. Dern. Ars..

[27] Cui H., Ding X., Li W., Chen H., Li H. (2019). The Neutrophil Percentage to Albumin Ratio as a New Predictor of In-Hospital Mortality in Patients with ST-Segment Elevation Myocardial Infarction. Med. Sci. Monit..

[28] Sun T., Shen H., Guo Q., Yang J., Zhai G., Zhang J., Zhang B., Ding Y., Cai C., Zhou Y. (2020). Association between Neutrophil Percentage-to-Albumin Ratio and All-Cause Mortality in Critically Ill Patients with Coronary Artery Disease. Biomed. Res. Int..

[29] Yu Y., Liu Y., Ling X., Huang R., Wang S., Min J., Xiao J., Zhang Y., Wang Z. (2020). The Neutrophil Percentage-to-Albumin Ratio as a New Predictor of All-Cause Mortality in Patients with Cardiogenic Shock. Biomed. Res. Int..

[30] Sorci-Thomas M.G., Thomas M.J. (2016). Microdomains, Inflammation, and Atherosclerosis. Circ. Res..

[31] Milan Manani S., Virzì G.M., Clementi A., Brocca A., de Cal M., Tantillo I., Ferrando L., Crepaldi C., Ronco C. (2016). Pro-Inflammatory Cytokines: A Possible Relationship with Dialytic Adequacy and Serum Albumin in Peritoneal Dialysis Patients. Clin. Kidney J..

[32] Ritchie R.F., Palomaki G.E., Neveux L.M., Navolotskaia O., Ledue T.B., Craig W.Y. (1999). Reference Distributions for the Negative Acute-Phase Serum Proteins, Albumin, Transferrin and Transthyretin: A Practical, Simple and Clinically Relevant Approach in a Large Cohort. J. Clin. Lab. Anal..

[33] Mikhailidis D.P., Ganotakis E.S. (1996). Plasma Albumin and Platelet Function: Relevance to Atherogenesis and Thrombosis. Platelets.

[34] Naruko T., Ueda M., Haze K., van der Wal A.C., van der Loos C.M., Itoh A., Komatsu R., Ikura Y., Ogami M., Shimada Y. (2002). Neutrophil Infiltration of Culprit Lesions in Acute Coronary Syndromes. Circulation.

[35] Baetta R., Corsini A. (2010). Role of Polymorphonuclear Neutrophils in Atherosclerosis: Current State and Future Perspectives. Atherosclerosis.

[36] American Diabetes Association 2 (2019). Classification and Diagnosis of Diabetes: Standards of Medical Care in Diabetes-2019. Diabetes Care.

[37] Williams B., Mancia G., Spiering W., Rosei E.A., Azizi M., Burnier M., Clement D.L., Coca A., de Simone G., Dominiczak A. (2018). 2018 ESC/ESH Guidelines for the Management of Arterial Hypertension. Eur. Heart J..

[38] Civeira F., Arca M., Cenarro A., Hegele R.A. (2022). A Mechanism-Based Operational Definition and Classification of Hypercholesterolemia. J. Clin. Lipidol..

[39] Gibson C.M., de Lemos J.A., Murphy S.A., Marble S.J., McCabe C.H., Cannon C.P., Antman E.M., Braunwald E., TIMI Study Group (2001). Combination Therapy with Abciximab Reduces Angiographically Evident Thrombus in Acute Myocardial Infarction: A TIMI 14 Substudy. Circulation.

[40] Kurtul A., Yarlioglues M., Celik I.E., Duran M., Elcik D., Kilic A., Oksuz F., Murat S.N. (2015). Association of Lymphocyte-to-Monocyte Ratio with the No-Reflow Phenomenon in Patients Who Underwent a Primary Percutaneous Coronary Intervention for ST-Elevation Myocardial Infarction. Coron. Artery Dis..

[41] Yu L., Chen J., Zhang J. (2024). Meta-Analysis of the Correlation between Inflammatory Response Indices and No-Reflow after PCI in Patients with Acute STEMI. Am. J. Transl. Res..

[42] Shumakov D.V., Shekhyan G.G., Zybin D.I., Yalymov A.A., Vedenikin T.Y., Popov M.A. (2021). In-stent restenosis: Symptoms, hemodynamic signs, pathogenesis and treatment. Russ. Cardiol. Bull..

[43] Wang L., Huang S., Zhou Q., Dou L., Lin D. (2024). The Predictive Value of Laboratory Parameters for No-Reflow Phenomenon in Patients with ST-Elevation Myocardial Infarction Following Primary Percutaneous Coronary Intervention: A Meta-Analysis. Clin. Cardiol..

[44] Wong D.T.L., Puri R., Richardson J.D., Worthley M.I., Worthley S.G. (2013). Myocardial ‘No-Reflow’—Diagnosis, Pathophysiology and Treatment. Int. J. Cardiol..

[45] Tian J., Liu Y., Liu Y., Song X., Zhang M., Xu F., Yuan F., Lyu S. (2017). Prognostic Association of Circulating Neutrophil Count with No-Reflow in Patients with ST-Segment Elevation Myocardial Infarction Following Successful Primary Percutaneous Intervention. Dis. Markers.

[46] Yin R., Zhu W., Chen W., Shen J., Wu Y., Wang Z. (2025). The Relationship between Neutrophil Percentage-to-Albumin Ratio and Slow and Normal Coronary Flow Phenomenon. BMC Cardiovasc. Disord..

[47] Karasu M., Karaca Y., Yıldırım E., Kobat M.A., Er F. (2023). Neutrophil-to-Albumin Ratio: A Promising Tool for CAD Assessment in Non-ST Elevation AMI. Eur. Rev. Med. Pharmacol. Sci..

[48] Zhao M., Huang X., Zhang Y., Wang Z., Zhang S., Peng J. (2024). Predictive Value of the Neutrophil Percentage-to-Albumin Ratio for Coronary Atherosclerosis Severity in Patients with CKD. BMC Cardiovasc. Disord..

[49] Serhatlioglu F., Yilmaz Y., Baran O., Yilmaz H., Kelesoglu S. (2025). Inflammatory Markers and Postoperative New-Onset Atrial Fibrillation: Prognostic Predictions of Neutrophil Percent to Albumin Ratio in Patients with CABG. Diagnostics.

[50] Karaca M., Gumusdag A. (2024). Prognostic Role of Neutrophil Percentage-to-Albumin Ratio in Patients with Non-ST-Elevation Myocardial Infarction. Medicina.

[51] Pinheiro Machado G., Araujo G.N., Carpes C.K., Lech M.C., Mariani S., Valle F.H., Bergoli L.C.C., Wainstein R.V., Wainstein M.V. (2019). Elevated Neutrophil-to-Lymphocyte Ratio Can Predict Procedural Adverse Events in Patients with ST-Elevation Myocardial Infarction Undergoing Primary Percutaneous Coronary Intervention. Coron. Artery Dis..

[52] Matteucci A., Bonanni M., Versaci F., Frati G., Peruzzi M., Sangiorgi G., Biondi-Zoccai G., Massaro G. (2022). Cardiovascular Medicine: A Year in Review. Minerva Cardiol. Angiol..

[53] Arques S. (2018). Human Serum Albumin in Cardiovascular Diseases. Eur. J. Intern. Med..

